# Proteome Mapping of Adult Zebrafish Marrow Neutrophils Reveals Partial Cross Species Conservation to Human Peripheral Neutrophils

**DOI:** 10.1371/journal.pone.0073998

**Published:** 2013-09-03

**Authors:** Sachin Kumar Singh, Sachin Sethi, Sriram Aravamudhan, Marcus Krüger, Clemens Grabher

**Affiliations:** 1 Institute of Toxicology and Genetics, Karlsruhe Institute of Technology, Karlsruhe, Germany; 2 Max Planck Institute for Heart and Lung Research, Bad Nauheim, Germany; Federal Institute for Vaccines and Biomedicines, Germany

## Abstract

Neutrophil granulocytes are pivotal cells within the first line of host defense of the innate immune system. In this study, we have used a gel-based LC-MS/MS approach to explore the proteome of primary marrow neutrophils from adult zebrafish. The identified proteins originated from all major cellular compartments. Gene ontology analysis revealed significant association of proteins with different immune-related network and pathway maps. 75% of proteins identified in neutrophils were identified in neutrophils only when compared to neutrophil-free brain tissue. Moreover, cross-species comparison with human peripheral blood neutrophils showed partial conservation of immune-related proteins between human and zebrafish. This study provides the first zebrafish neutrophil proteome and may serve as a valuable resource for an understanding of neutrophil biology and innate immunity.

## Introduction

Zebrafish have become a powerful vertebrate model system for the study of hematopoiesis and immunity [1]. The use of zebrafish to study the ontogeny of leukocyte subsets [2], immune cell migration [3], regeneration [4], [5], and host-pathogen interactions [6] has provided new insights towards the understanding of innate immunity in the developing vertebrate embryo. A particular advantage of the zebrafish system lies in the chronological separation of innate and adaptive immunity during embryonic and larval development [7]. Only the innate immune system is active during larval stages, whereas the development of a functional adaptive immune system requires several weeks [8]. Neutrophil granulocytes and primitive macrophages are the most abundant leukocytes in zebrafish larvae and are the primary component of innate immunity, to provide the first line of defense against infection or injury. Tissue damage or infection stimulates immediate neutrophil recruitment to the site of trauma [3], [4]. Neutrophils at the scene may fight noxious microbes by the release of granular content. Moreover, neutrophils have the potential to initiate other parts of the immune system including an adaptive immune response through the production of pro-inflammatory cytokines and chemokines [9].

Modern mass spectrometry allows for cell type, or tissue-specific identification and quantification of proteins [10] and several studies have been performed to explore the proteome of neutrophils from different animal model systems [11]. Recently, various proteome analyses have also been done on zebrafish tissue. For example, the protein content of adult zebrafish brain [12], caudal fin [13]–[15], kidney [16], liver [17] and gill [18] as well of developing zebrafish embryos [19] has been explored. However, although the zebrafish is increasingly applied for immunity-related research, a resource for the protein composition of neutrophils or other immune cells of zebrafish was still lacking.

In this study, we performed a comprehensive analysis of the proteome of neutrophils from the whole kidney marrow, the major hematopoietic compartment, of adult zebrafish. We employed a gel-based LC-MS/MS approach to explore the proteome of fluorescently labeled primary neutrophils and identified 1544 proteins. In an attempt to narrow down the protein content that may be specific to neutrophils, we further compared our data with previously reported protein data from other tissue types. Subsequently, we performed a comparative analysis between the identified neutrophil proteome of zebrafish and publicly available data from human neutrophil proteome investigations in order to evaluate the degree of cross-species conversation. Due to the lack of available protein data from human marrow neutrophils, we compared zebrafish marrow neutrophils with human peripheral neutrophils. Our data set thus expands on previous neutrophil proteome data from other species and serves as a valuable resource towards a better understanding of zebrafish neutrophil biology and innate immunity.

## Materials and Methods

### Zebrafish Lines

We used the neutrophil-specific zebrafish reporter line *Tg(lyzC:DsRed)^nz50^* [20]. All zebrafish husbandry and experimental procedures were performed in accordance with the German animal protection standards (Animal Protection Law, BGBl. I, 1934 (2010)) and were approved by the Local Government of Baden-Württemberg, Regierungspräsidium Karlsruhe, Germany (License number: Proteome analyses of adult zebrafish: Az.: 35–9185.81/G-170/12 and general license for fish maintenance and breeding: Az.: 35–9185.64).

### Isolation of Marrow Neutrophils

Twenty adult *Tg(lyzC:DsRed)* zebrafish were anesthetized with 0.2 mg/ml of Tricane and killed in an ice bath. After decapitation and ventral incision the organs were removed to expose the whole kidney marrow (WKM). WKM was then removed and placed in L-15 media (Sigma-Aldrich) with 5% FBS. The WKM was triturated and subsequently passed through a 40 micron filter and centrifuged at 450×g. Dissociated cells were suspended in media and filtered again. The single cell suspension was analyzed and sorted on a FACS Aria II flow cytometer (BD Biosciences). The WKM cell suspension was gated based on size and granularity using forward and side scatter characteristics, respectively. The fluorescently labeled cells from the DsRed reporter line were visualized using the PE filter set (582/15 nm) upon excitation by a 488 nm laser line. DsRed positive cells were back-gated to confirm their myeloid characteristics and collected. Purity was verified by both, FACS reanalysis and visual inspection on a DM5500 fluorescent microscope (Leica). Cell viability was determined by Tryphan blue staining and cell numbers were determined using a hematocytometer. Subsequently, cells were snap-frozen and stored at −80°C for later proteomics analysis.

### 1D Electrophoresis

Sorted neutrophil cells (in duplicate) were lysed in protein lysis buffer containing 4% SDS, 100 mM Tris/HCl, pH 7.6 (Sigma) and heated at 99°C for 10 min. Subsequently, protein samples were homogenized by ultra-sonication (3 times, 30 second pulses with an interval of 60 seconds). The protein supernatant was collected after centrifugation at 15000 g for 15 minutes at room temperature, and total protein concentration was measured using the Bradford method. Fifty micrograms of protein were subjected in duplicate to gel electrophoresis using precast 4–12% Nu-PAGE gradient gels (Invitrogen) and separated on the basis of their molecular weight. The gel was stained with Colloidal Blue Staining Kit (Invitrogen) overnight. Subsequently, the gel was de-stained and documented. Gel lanes were cut into 9 slices, and each slice was de-stained by washing with 50 mM ammonium bicarbonate/50% ethanol followed by absolute ethanol. This was followed by reduction and alkylation with DTT and iodacetamide, respectively. Subsequently, gel slices were digested with mass-spectrometry grade trypsin with an enzyme to protein ration of 1∶100. Peptides were eluted from the gel pieces using acetonitrile and desalted using homemade C18 columns (stage tips) [21].

### LC-MS/MS Analysis

Each trypsin-digested sample, representing the peptide content of one gel piece was eluted from stage tips, subjected to an automatic sampler and further analyzed by nano-reversed phase chromatography using an Agilent 1100 nanoflow system, online-coupled via in house packed fused silica capillary column emitters (length 15 cm; ID 75 µM; resin ReproSil-Pur C18-AQ, 3 µm), and a nanoelectrospray source (Proxeon) to a LTQ Orbitrap XL mass spectrometer (Thermo Scientific). Linear gradients from 5–35% buffer B (80% acetonitrile, 0.5% acetic acid) over 150 minutes at 200 nl/min were applied to elute peptides from the C18 column. The whole mass spectrometry process was operated in data-dependent mode, collecting collision-induced MS/MS spectra from LTQ-FT full scans from m/z 300 to m/z 1800; resolution r = 60,000; LTQ isolation and fragmentation at a target value of 10000. AGC target MS 30000 and 100 ms and 300–750 ms maximum injection time were applied for ion trap and Orbitrap, respectively. Subsequently, a 1.2 Dalton ion selection window for MS/MS was applied. The five most intense peaks from full MS scan were fragmented in a linear ion trap using CID (35% normalized collision energy) and for LTQ Orbitrap measurements (MS/MS), 15 most intense peaks were selected for fragmentation in the linear ion trap.

### Data Analysis

The acquired mass spectrometry raw data was further analyzed by MaxQuant software (1.3.0.5). Peptide identification was performed by searching the peak list against the international protein index sequence database (zebrafish IPI, version 3.54) supplemented with commonly observed contaminants (embedded in MaxQuant) and concatenated with reversed versions of all sequences. Carbomidomethylation of cysteine was set as fixed modification; oxidation of methionine was kept as variable modification. Additionally, the search parameters included use of proteolytic enzyme and up to a maximum of 2 missed cleavages. Peptide mass tolerance was 6 ppm for precursor ion and 0.5 Dalton for fragment ion. A false discovery rate of 1% was applied for protein and peptide identification and proteins identified with at least 2 peptides or a single unique peptide were incorporated for data analysis.

### Gene Ontology Analysis

Identified neutrophil proteins were further analyzed for their cellular localization, biological processes and molecular functions using STRAP (Software Tool for Rapid Annotation of Proteins software program) analysis [22]. Additionally, gene IDs of identified proteins were obtained using DAVID (Database for Annotation Visualization and Integrated Discovery) analysis [23]. Subsequently, the GeneGo software (www.genego.com) was applied to establish process, network and pathways maps with identified proteins.

## Results

Upon analysis of total protein extract (50 µg) from adult zebrafish marrow neutrophils by 1DE and LTQ Orbitrap XL MS, a total of 1544 proteins were identified from 9 trypsin-digested fractions (Table S1a & 1b). About 78% (1204/1544) of the proteins were identified upon the presence of multiple peptides. The remaining 340 proteins were identified on the basis of unique single peptides (Table S1c). Protein abundance ranged from ion intensities of 10^10^ to 10^4^, with most abundant proteins represented by Mpx, Actin, Histone 2B and L-plastin (ion intensity 1×10^10^–4.9×10^9^) and the least abundant protein was Dhrs7 (ion intensity 10^4^). Out of the 1544 proteins identified, 201 were largely uncharacterized proteins (Table S1d) (with zgc or loc identifiers), while 195 proteins lacked annotated gene symbols and were thus identified through IPI IDs (Table S1e).

**Figure 1:**
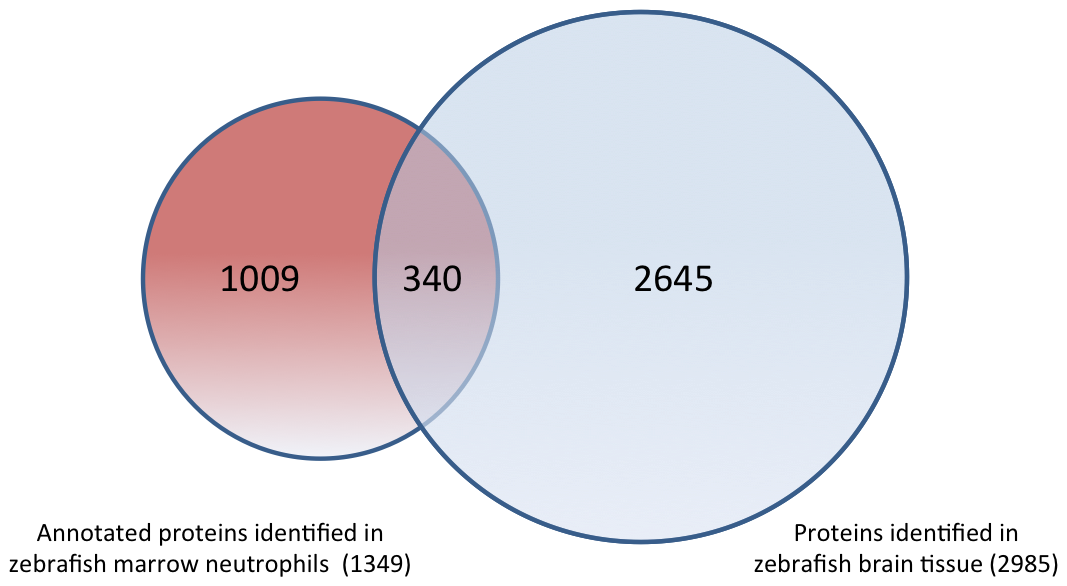
Zebrafish brain and neutrophil proteome comparison. Venn diagram comparing zebrafish whole brain and zebrafish neutrophil proteome data sets.

The protein content of a cell largely determines its functional state and may even provide a better indication of cellular function than the transcriptome. Nevertheless, highly abundant proteins often hinder the identification of other, less abundant proteins that may be more specific to the tissue- or cell type under investigation. To narrow down the protein content that may be specific to neutrophils versus other tissues we compared our neutrophil data set with a protein data set from other tissue devoid of neutrophils. Hence, we performed a comparison of the adult zebrafish brain proteome [24] with the current set of 1349 proteins with annotated gene symbols from neutrophils (Table S1f). Comparison of the neutrophil proteome with that of adult zebrafish brain revealed that 25% (340/1349) of proteins identified in neutrophils were also present in tissue from adult brain (Figure 1 & Table S2a). These common proteins included many cytoskeletal, enzymatic and metabolic proteins. The remaining 75% were enriched in neutrophils versus adult zebrafish brain tissue (Table S2b). A list of the most abundant neutrophil-specific proteins is shown in Table 1.

**Table 1:** Most abundant proteins in zebrafish neutrophils.

Gene Name	Protein name	Uniprot Id	Intensity	No. of peptides
npsn	Nephrosin	Q503K7	6974900000	13
lcp1;pls2	Plastin-2	Q6P698	4951600000	45
h2afx	similar to histone cluster 2	Q7ZUY3	4560900000	9
lyz	Lysozyme C	Q24JW2	3172600000	17
lect2	Leukocyte cell-derived chemotaxin 2 like	Q0H0R9	2321700000	14
nccrp1	nonspecific cytotoxic cell receptor protein 1	A1L1Z5	1100500000	14
si:dkeyp-46h3.6	Histone H3	Q4QRF4	1019300000	7
prdx5	Peroxiredoxin 5	Q502C8	988140000	9
cap1	Adenylyl cyclase-associated protein	A7E2H8	811930000	22
coro1a	Coronin, actin binding protein, 1A	Q7SX58	626720000	18
rdx	Radixin;67 kDa protein	Q66I42	565640000	31
anxa3b	annexin A3b	A8E5E5	463900000	26
wdr1	WD repeat domain 1	Q6NY25	342040000	24
calrl	Calreticulin like	Q6DI13	301730000	25
h1fx	H1 histone family, member X	Q802U8	240540000	4
rab1a	RAB1A	Q7ZSZ0	238780000	8
clic1	Chloride intracellular channel 1	Q6NYF2	237250000	13
rps13	Ribosomal protein S13	Q6IMW6	234040000	9
arpc3	Actin related protein	Q6ZM62	224530000	9
nme2	Nucleoside diphosphate kinase	Q7SXG5	213480000	7

List of the 20 most abundant neutrophil-specific proteins identified in zebrafish marrow neutrophils. Protein abundance is shown as ion intensity. Number of peptides depicts the number of individual peptides identified for each protein.

### Process and Pathway Analysis

**Figure 2:**
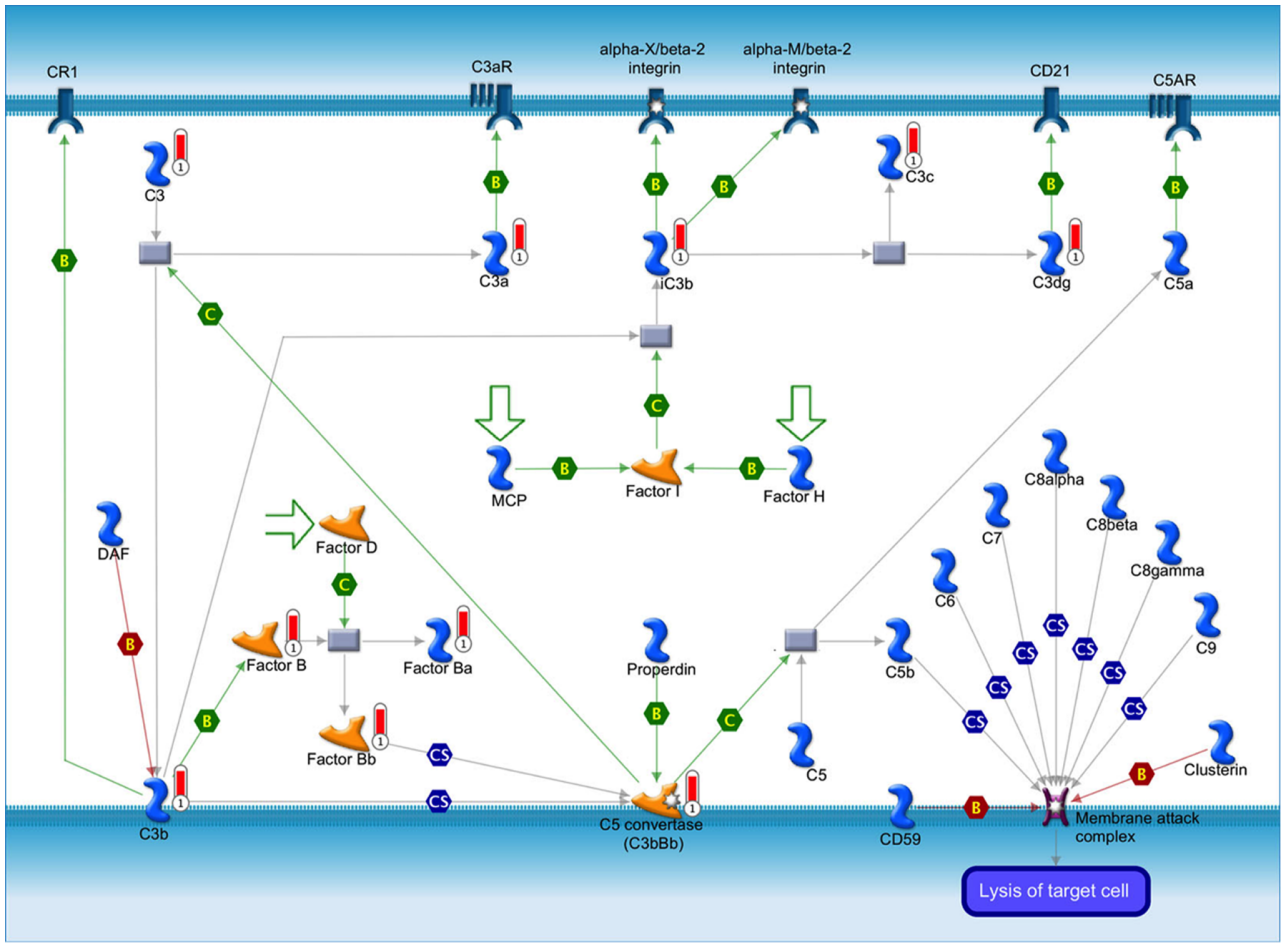
Complement pathway map analysis of zebrafish neutrophil proteins. Alternative complement pathway map identified from the zebrafish neutrophil proteome. 10 different zebrafish neutrophil proteins were found associated with the alternative complement pathway. Proteins with red color thermometer bar represent the zebrafish neutrophil proteins identified in this study.

To determine annotated functional roles of the proteins identified from neutrophils, 1009 proteins with recognized gene symbols were submitted to Gene-Go (Metacore) for functional and network pathways analysis. Oxidative phosphorylation and immune response, including alternative complement pathways, chemotaxis and CXCR4 signaling, together with cytoskeletal remodeling, apoptosis and survival, and transport were among the 20 most prominently associated pathways (Figure S1). For example, C3, C3a, iC3b, C3c, C3dg, C3b, C5 convertase as well as factor B, Bp, Ba were among the identified proteins, which were significantly associated with the humoral branch of the innate immune system acting to protect the host from microorganisms (Figure 2) [25].

**Figure 3:**
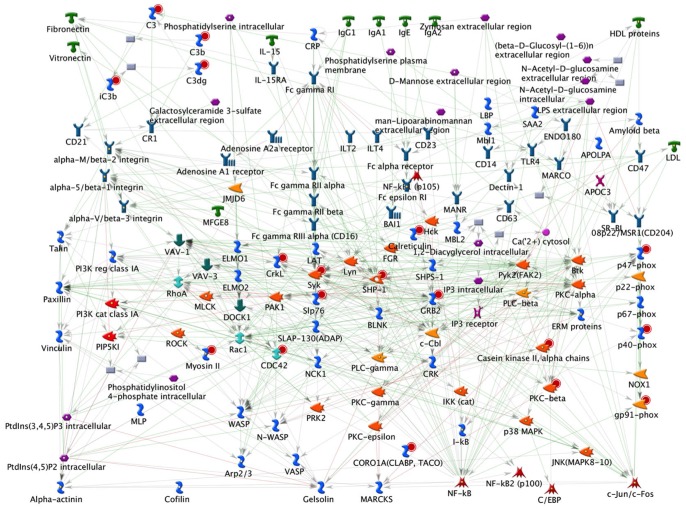
Phagocytosis network process analysis of zebrafish neutrophil proteins. Phagocytosis process network identified from the neutrophil proteome dataset. 18 neutrophil proteins were found associated in this pathway. Proteins represented with a red circle represent the proteins identified in this study.

In addition, global process network analysis revealed a significant association of identified proteins with 12 different process networks (Figure S2). A total of 67 proteins were associated with translation (initiation, elongation, termination) and 28 proteins with transcription (mRNA processing). 48 proteins were associated with immune response (30 with phagosome antigen presentation and 18 with phagocytosis). Phagocytosis is a key process of the innate immune system, in which cells engulf foreign particles or cell debris. For example, iC3b, C3, Myosin, Shp −1, Syk, Crkl, CDC42, Slp 76 and Hck and others are commonly associated with phagocytosis (Figure 3) [26]. Further, enrichment by protein function analysis mapped the identified proteins into six different functional groups including enzymatic proteins, proteases, kinases, transcription factors, ligands and receptors (Table 2).

**Table 2:** Functional categories of zebrafish neutrophil proteins.

Classification	Gene symbol
Proteases	Acp1, Ppp3cc,Ptpn6, Mtmr6,Ppp1cb
Transcripation factors	Drap1, Pa2g4, Gabpa, Hmgb1, Hcfc1
Receptors	Atrn, M6pr, Ptgrc, CD41, CD82
Ligands	Lect2, Mif, Stoml2, B2m, Manf
Enzymes	Arsa, Asah1, Bdh1, Hexb, Glb1
Kinases	Csnk1a1i, Cpne3, Prkib, Slk
Phosphatases	Ppap2a, Minpp1, Nudt5, Pgaam1, Fbp1

List of the five most abundant proteins of different functional categories in zebrafish neutrophils.

### Cross-Species Correlation

**Figure 4:**
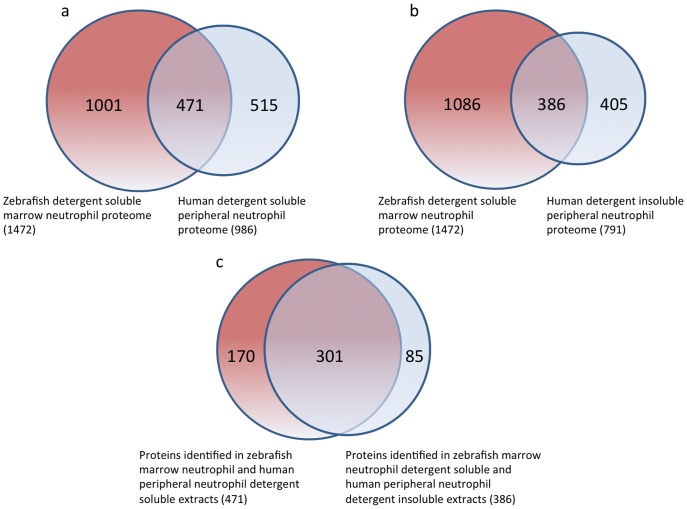
Cross species comparison of zebrafish and human neutrophil proteomes. Venn diagrams comparing the zebrafish and human neutrophil proteomes. Comparison of zebrafish and human detergent soluble proteomes (a). Comparison of zebrafish detergent soluble and human detergent insoluble proteomes (b). A majority of proteins common between zebrafish detergent soluble and human detergent insoluble are also present in the human detergent soluble data set (c).

In lieu of protein data from human marrow neutrophils, we compared our dataset with a recently reported data of a whole cell lysate proteome of neutrophils isolated from peripheral blood [27]. Of the 1544 proteins identified from neutrophils of adult zebrafish, only 1148 proteins were annotated with unique gene symbols (Table S2c). To maximize the number of proteins available for comparison we blasted the zebrafish proteins without unique gene symbols (395) against the human protein database at NCBI to identify homologous proteins. Out of the 395 proteins, 89 produced hits with greater than 80% identity, 102 with 60–80% identity and 133 proteins produced hits with 40–60% identity to human proteins (Table S3a). Including the proteins identified through blast analysis (Table S3b), we were able to compare 1472 zebrafish proteins with the human dataset. Tomazella et al. identified 1249 proteins, where 430 proteins were identified only from detergent soluble extracts and 240 proteins were identified only from detergent insoluble extracts of human neutrophils. 579 proteins were identified from both sample preparations. The proteome data of the here discussed zebrafish neutrophils are based on detergent soluble extracts. We therefore compared the detergent soluble human and detergent soluble zebrafish neutrophil proteome data sets based on gene symbol. More than 47% (471) of all proteins identified in the detergent soluble human neutrophil proteome were also found in the zebrafish neutrophil proteome (Figure 4a & Table S3c). Surprisingly, comparison of the detergent insoluble proteome of human neutrophils and the zebrafish detergent soluble neutrophil proteome also revealed a more than 48% (386) overlap of between the human and zebrafish sample preparations (Figure 4b). However, we found that 78% (301/386) of these proteins, were found in both, the detergent soluble and detergent insoluble human neutrophil proteome data set (Figure 4c).

To perform cross-species comparison in gene ontology, the 471 proteins common between human and zebrafish detergent soluble extracts and 1002 (Table S3d) proteins found in zebrafish only but not in human neutrophils were submitted to Gene Go Metacore for functional, pathway and network analysis. Network process analysis revealed that commonly identified proteins significantly associate with 47 different network pathways, where six immune-related pathways were among the top 20 pathways (Figure S3). These include phagosome in antigen presentation, antigen presentation, neutrophil activation, amphoterin signaling, TCR signaling, TREM 1 signaling.

In contrast, non-common proteins were significantly associated with only 20 different network pathways, with high ranking pathways such as translation, transcription, cytoskeleton regulation, cell cycle and muscle contraction, including only two immune–related pathways (rank 10 and 11) (Figure S4).

Additionally, to investigate the conservation of immune related proteins between zebrafish and human neutrophils, Gene-Go Metacore analysis of both data sets was performed individually. Subsequently, the proteins specifically associated with immune system pathways were extracted and compared, revealing 54% similarity between zebrafish marrow neutrophils and human peripheral blood neutrophils. Although, proteins in both, human and zebrafish neutrophils were identified from all major compartments, the distribution to various compartments differed between the samples. In zebrafish neutrophils the percentage of nuclear proteins and proteins involved in macromolecular processes was higher than in human neutrophils. On other hand proteins from human neutrophils contained a higher proportion of proteins from plasma membrane, endosome, cytoplasm and mitochondria (Table 3a). Furthermore, characterization based on biological processes revealed that in zebrafish almost twice as many proteins were associated with cellular processes compared to human neutrophils. Similarly, the fraction of proteins associated with metabolic processes was higher in zebrafish than in human neutrophils. Whereas, the numbers of proteins involved in regulation and development were similar in both species. However, human neutrophils from peripheral blood contained more proteins that are specifically associated with response to stimulus immune system processes and interaction with cells and organism than marrow neutrophils from zebrafish (Table 3b).

**Table 3:** Comparison of human and zebrafish neutrophil proteomes based on subcellular localization (a) and on association with biological process (b).

Subcellular localization (a)		
Compartment	Human	Zebrafish
Cytoplasm	19%	19%
Macromolecular complex	5%	10%
Intracellular organelles	10%	3%
Extracellular	6%	2%
Cell surface	1%	–
Plasma membrane	11%	3%
Cytoskeleton	6%	7%
Peroxisome	1%	–
Nucleus	13%	17%
Mitochondria	7%	5%
ER	4%	4%
Ribosome	–	8%
Chromosome	2%	3%
Endosome	4%	1%
Others	11%	19%
**Biological process (b)**		
**Process**	**Human**	**Zebrafish**
Regulation	21%	19%
Reproduction	3%	–
Response to stimulus	7%	3%
Cellular process	27%	44%
Developmental process	6%	7%
Growth	2%	–
Immune system process	5%	1%
Interaction with cells and organism	7%	2%
Localization	6%	6%
Metabolic process	8%	11%
Other	12%	8%

## Discussion

As in humans, neutrophils are the most abundant innate immune cells also in adult zebrafish. The application of proteomics for the characterization of neutrophils bears strong potential towards a better understanding of neutrophil biology. In this study, we performed a comprehensive analysis of the proteome of neutrophils from the whole kidney marrow, the major hematopoietic compartment, of adult zebrafish. We employed a gel-based LC-MS/MS approach to explore the proteome of FACS sorted neutrophils and identified 1544 proteins expressed in neutrophils. Subsequently, to identify proteins that may be particularly enriched in neutrophils, we compared our data with the only publicly available proteome data of healthy brain of zebrafish. The healthy brain is expected to be devoid of neutrophils and the immune component present in the brain is comprised of microglia only [28]. The proteome composition of wild zebrafish may not be entirely identical to the laboratory strain used in this study. However, the comparison with non-granulocytic tissue revealed that more than 75% of the proteins were enriched in neutrophils versus zebrafish brain tissue. Further, functional characterization of these proteins revealed that the majority of these proteins belong to various immune-related pathways such as alternative complement pathways, chemotaxis and CXCR4 signaling and inflammation.

In order to evaluate the degree of cross-species conservation, we performed a comparative analysis between the identified neutrophil proteome of zebrafish and a recently published data set of human neutrophils [27]. Of 986 proteins identified from the detergent soluble fraction of the human neutrophil proteome, only 47% (471) proteins were also present in the zebrafish neutrophil data set evaluated in this study. As expected, proteins common between human and zebrafish display significant association with various distinctly immune-related pathways including neutrophil activation, antigen presentation and phagocytosis. Interestingly, 386 proteins were also found to be common between the zebrafish neutrophil proteome and the human detergent insoluble neutrophil proteome. However, only 85 proteins were actually specific to the human detergent insoluble neutrophil proteome, while the majority of proteins identified in the detergent insoluble fraction were also present in the detergent soluble fraction of human neutrophils. Analysis of the annotated subcellular localization of these proteins showed that only about 2% are membrane associated proteins, while the majority were cytoplasmic or nuclear proteins indicating technical rather than biological reasons for the overlap of proteins between detergent soluble and detergent insoluble fractions. Among proteins conserved in zebrafish and human were many with well-characterized roles in the immune system such as L-plastin, Ferritin and S100. For example, Ferritin plays a pivotal role in iron homeostasis [29] and restricts iron availability to microorganisms [30]. The S100 protein is a calcium-binding protein [31] involved in various processes of the immune system such as leukocyte chemotaxis and adhesion [32], [33]. Moreover, we also identified several members of the Rab protein family, heat shock proteins and histones. Rab proteins are known to regulate membrane trafficking. For example, Rab 5 plays a significant role in chemoattractant receptor endocytosis and fusion of intracellular granules with phagosomes in human neutrophils [34]. Similarly, some members of the HSP family such as HSP60, HSP70, HSP90 stimulate the cells of innate immune system and thus act as danger signaling molecules during inflammation [35]. Particular histones, along with DNA and antimicrobial proteins, are an important component of neutrophil extracellular trap (NETs) [36]. Moreover, we have also identified several proteins such as WD repeat domain 1 (Wdr1), sorting nexin (Snx5), sorcin (Sri), which functions are not well established in immune defense but may nevertheless be critical. For example, sorcin is a calcium binding protein involved in intracellular Ca^2+^ homeostasis and is expressed in leukocytes and lymphocytes [37]. Sorcin may thus be important for inflammation though its calcium regulatory function as calcium is an important mediator in innate immunity [38]. Similarly, Sorting nexin 5 is a intracellular trafficking protein and may play a vital role in modulation of secretory pathways for controlling cytokines and inflammation [39]. The Wdr 1 protein promotes cofilin-mediated actin filament disassembly [40]. Hence, Wdr1 may be important for immune cell migration during inflammation [41].

The non-conserved proteins on the other hand were typically associated with translation, transcription, cytoskeletal remodeling and cell cycle. Neutrophils are cells of the myeloid lineage originating from marrow tissue and residing as immature or inactive cells in bone marrow (mammalian) or kidney marrow (zebrafish). The human proteome data used in this study is derived from peripheral blood neutrophils, while the zebrafish proteome studied here represents marrow neutrophils. Therefore, the limited degree of conservation between the human and fish proteomes analyzed here may in part be due to the differing nature of the samples. Kidney marrow neutrophils consist of developing and mature neutrophils, while peripheral blood neutrophils consist of functionally mature neutrophils only. In addition, although many aspects of the human and zebrafish hematopoietic systems are similar, differences do exist. For example the human hematopoietic system contains three types of granulocytes, neutrophils, eosinophils and basophils. This distinction is less clear in zebrafish, where a distinct basophil type has not been characterized yet [42]. Thus it is conceivable that zebrafish neutrophils may combine functional repertoires that may be separated into multiple cell types in humans. Moreover, although we have no evidence that the lyzC^+^ granulocyte population used in this study contains other cell types than neutrophils, different maturation states may be present. Moreover, protein identification strongly depends on subcellular protein distribution and extraction method as well as sensitivity and precision of the mass-spectrometry approach used. Our data may suggest that our extraction method or MS approach yielded more nuclear proteins at the expense of membrane- and cell surfaces proteins as compared to the human data set. However, because similar extraction and buffer conditions as well as a similar MS approach were applied for both, human and zebrafish samples, the observed variations in subcellular protein distribution and content are likely due to differences in neutrophil differentiation, maturation or activation. Thus, deeper analyses of compartment-specific proteomes of matched sample types will likely result in higher yields and better comparability regarding cross-species conservation between zebrafish and human neutrophil proteomes.

Nevertheless, cross comparison of immune related proteins of zebrafish and human revealed a conservation of more than 54% between zebrafish and human proteome data sets. In light of the sample differences mentioned above, this degree of similarity suggests a considerable conservation between the two species and warrants more detailed analyses in the future. To further investigate the similarity between zebrafish and human innate immune systems on a functional level, it will be interesting to compare protein dynamics upon activation of zebrafish neutrophils through infection or upon sterile inflammation.

## Supporting Information

Figure S1 **Gene-Go pathway map analysis of zebrafish neutrophil proteins.** Most prominent Gene-Go pathway maps associated with identified neutrophil-specific proteins.
(TIF)

Figure S2 **Zebrafish neutrophil Gene-Go network process pathways.** Most prominent Gene-Go network process pathways associated with identified neutrophil-specific proteins.
(TIF)

Figure S3 **Cross-species neutrophil Gene-Go network process pathways.** Most prominent Gene-Go network process pathways associated with proteins identified in both zebrafish and human neutrophils.
(TIF)

Figure S4 **Gene-Go network process pathways of non-conserved neutrophil proteins.** Most prominent Gene-Go network process pathways associated with proteins that were not conserved between zebrafish and human neutrophils.
(TIF)

Table S1 a. List of all proteins identified in zebrafish neutrophils. b. List of associated identifier peptide sequences in zebrafish neutrophil**.** c. List of neutrophil proteins identified by unique single peptides. d. List of zgc and loc proteins identified in zebrafish neutrophils**.** e. List of zebrafish neutrophils proteins identified by IPI IDs**.** f. List of all annotated proteins identified in zebrafish neutrophils.
(XLSX)

Table S2 List of proteins common in zebrafish neutrophil versus brain proteomes. a. List of neutrophil-specific proteins (identified in zebrafish neutrophils but not in zebrafish brain tissue). b. List of proteins identified by unique gene name and Uniprot IDs.
(XLSX)

Table S3 a. Blast analysis of non-annotated zebrafish neutrophil proteins with human protein database. b. List of blasted proteins common between zebrafish and human neutrophil proteome. c. List of zebrafish neutrophil proteins common to human neutrophil whole cell lysate proteome. d. List of proteins not common between zebrafish and human neutrophil proteomes.
(XLSX)
