# Supplementary figures and images for: Proteome Mapping of Adult Zebrafish Marrow Neutrophils Reveals Partial Cross Species Conservation to Human Peripheral Neutrophils
Source: PLoS One. 2013 Sep 3;8(9):e73998. doi: 10.1371/journal.pone.0073998 (PMC3760823; doi:10.1371/journal.pone.0073998)

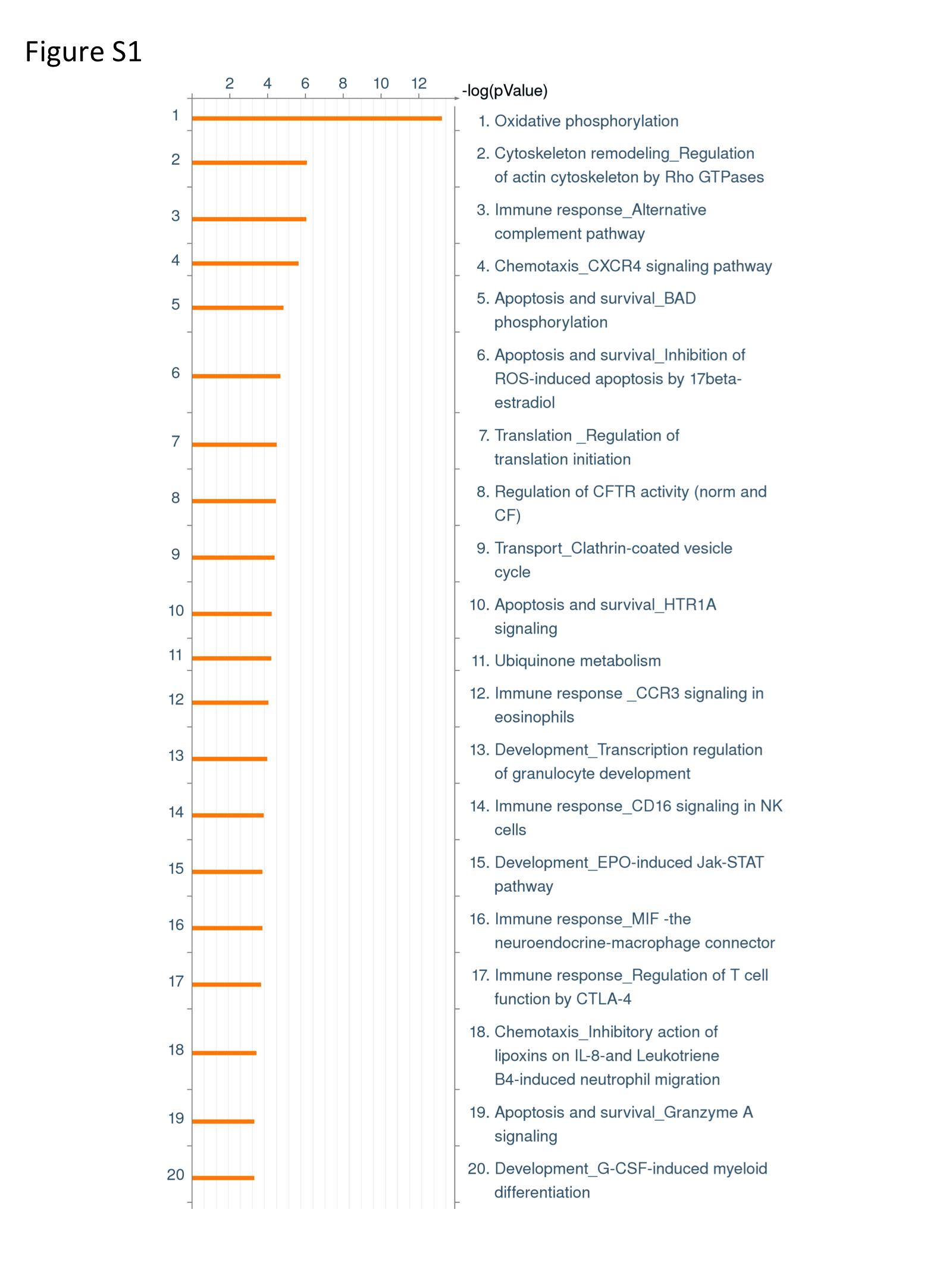

Supplement: Figure S1 — Gene-Go pathway map analysis of zebrafish neutrophil proteins. Most prominent Gene-Go pathway maps associated with identified neutrophil-specific proteins. (TIF) [file pone.0073998.s001.tif]

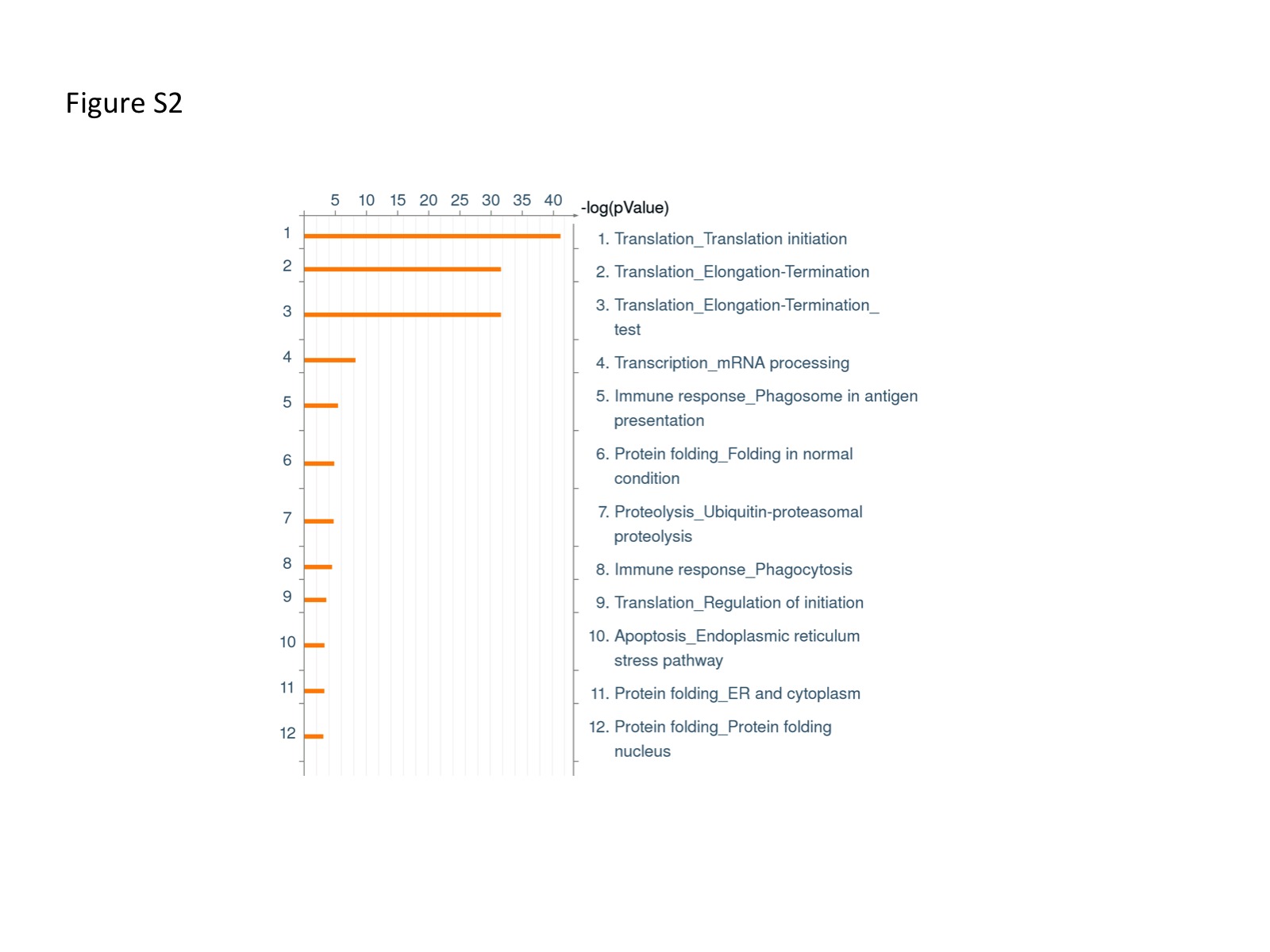

Supplement: Figure S2 — Zebrafish neutrophil Gene-Go network process pathways. Most prominent Gene-Go network process pathways associated with identified neutrophil-specific proteins. (TIF) [file pone.0073998.s002.tif]

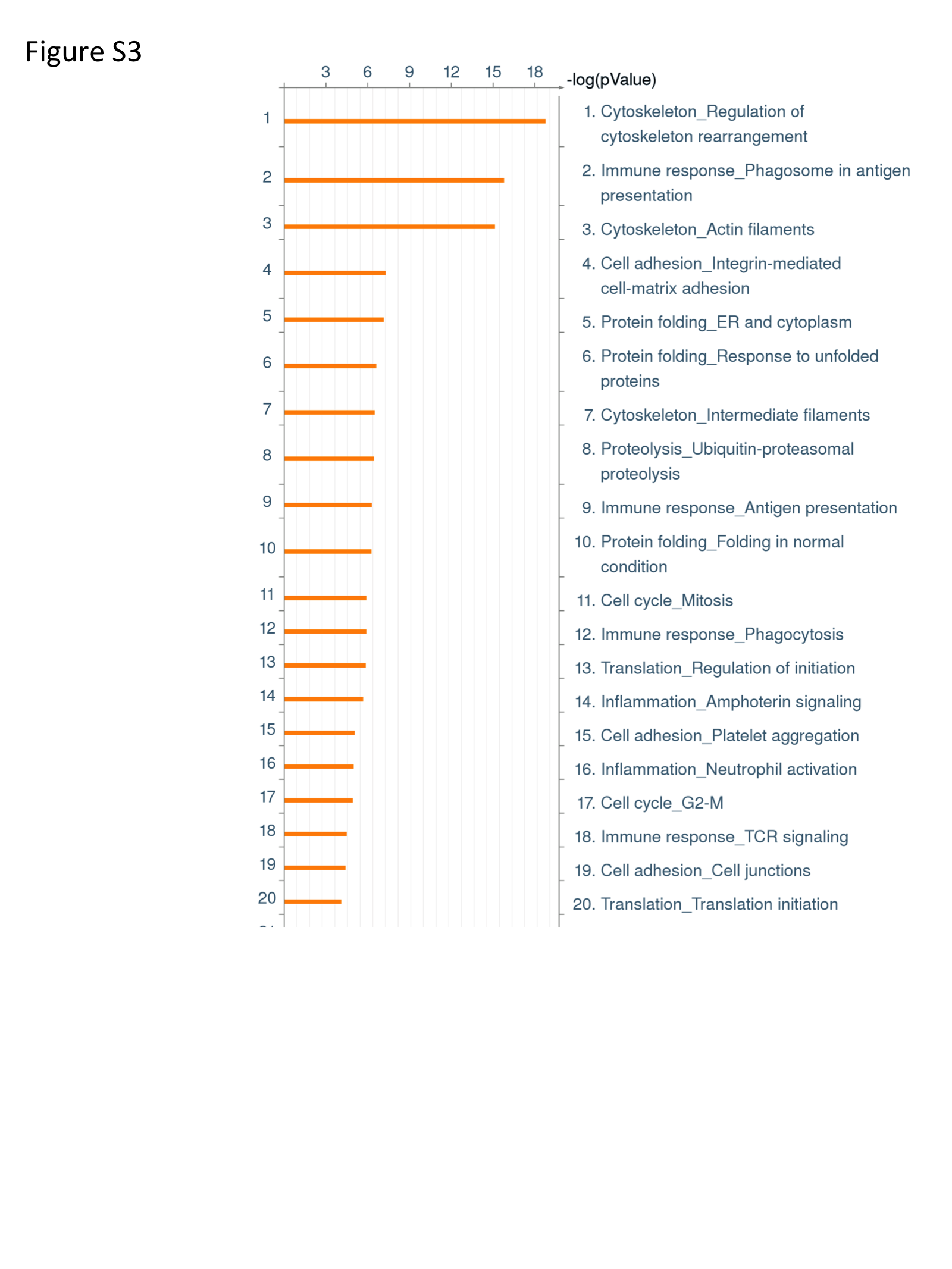

Supplement: Figure S3 — Cross-species neutrophil Gene-Go network process pathways. Most prominent Gene-Go network process pathways associated with proteins identified in both zebrafish and human neutrophils. (TIF) [file pone.0073998.s003.tif]

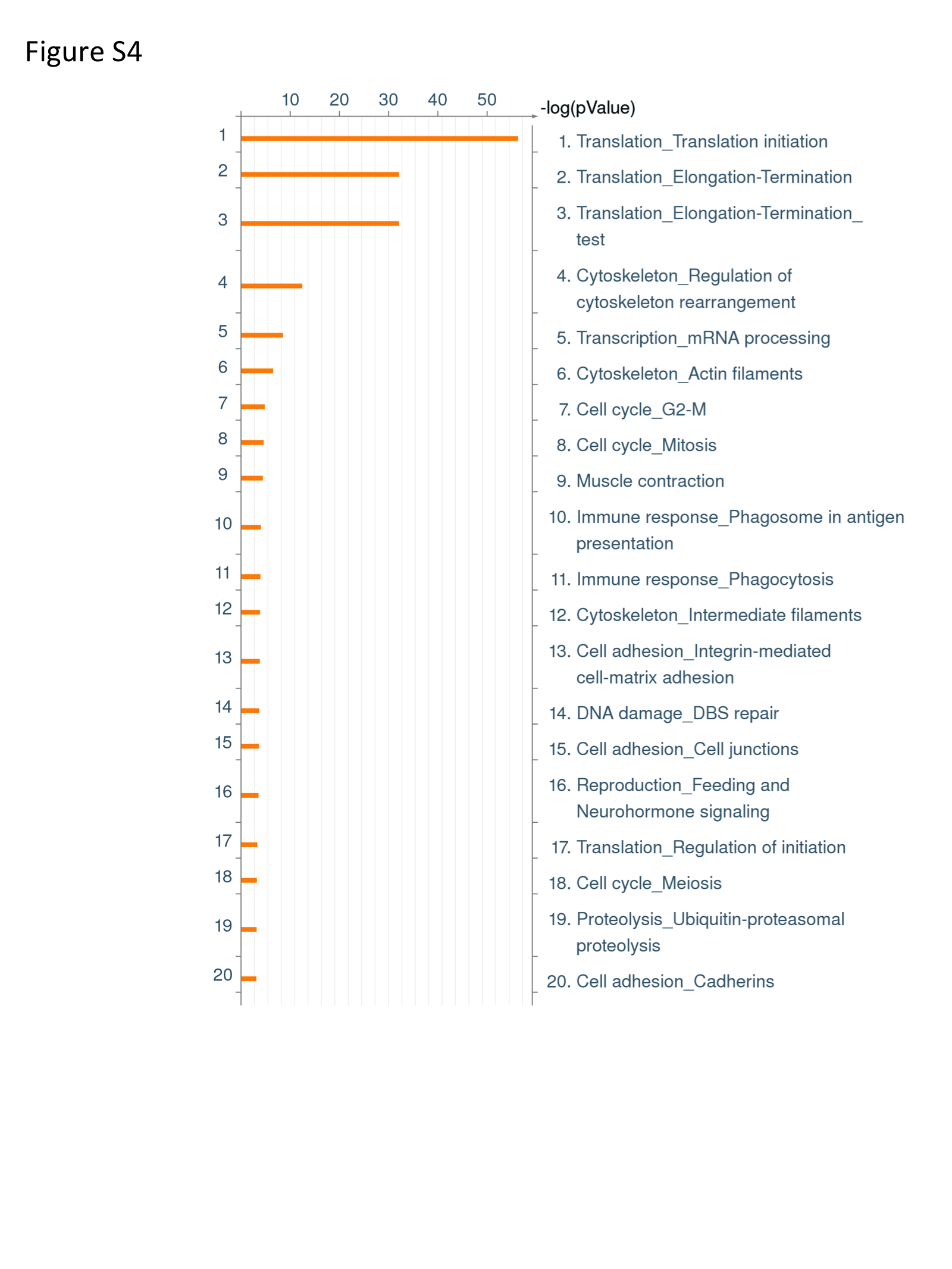

Supplement: Figure S4 — Gene-Go network process pathways of non-conserved neutrophil proteins. Most prominent Gene-Go network process pathways associated with proteins that were not conserved between zebrafish and human neutrophils. (TIF) [file pone.0073998.s004.tif]
